# A priming nudge targeting innovative farmers: A large-scale survey experiment

**DOI:** 10.1371/journal.pone.0345658

**Published:** 2026-03-26

**Authors:** Douadia Bougherara, Léa Gosset, Raphaële Préget, Sophie Thoyer

**Affiliations:** 1 CEE-M, Univ Montpellier, CNRS, INRAE, Institut Agro, Montpellier, France; 2 UMR MOISA, Univ Montpellier, CIRAD, Institut Agro, Montpellier, France; University of Hamburg: Universitat Hamburg, GERMANY

## Abstract

This article measures farmers’ innovativeness and the effectiveness of a priming nudge on their (stated) intention to adopt an innovation, namely the French “Low-carbon label” (LCL). The LCL is an innovative certification framework that provides farmers with a potential new “green business model,” enabling them to generate income through the sale of certified carbon credits earned by reducing their own greenhouse gas (GHG) emissions. Using 6,005 responses to an online survey with French farmers, we validate an original scale designed to measure farmers’ capacity to innovate and find that innovativeness is positively correlated with stated intention to adopt the LCL. We then evaluate with a randomized experiment included in the questionnaire the net impact of a priming nudge, defined as exposure to a lexical field designed to unconsciously activate psychological factors, and implemented here with references to innovation in order to target the most innovative farmers. We show that the nudge has no detectable impact on the surveyed sample: it neither increases adoption intentions among the most innovative farmers nor discourages the less innovative ones. This absence of effect leads us to discuss the effectiveness of nudges when trying to influence farmers’ high-stakes decisions.

## 1 Introduction

Rogers’ innovation adoption and diffusion model [1–2] has inspired a great deal of research in sociology and economics, notably on the diffusion of technological innovations in the agricultural sector and the dynamics of farmer adoption [3–4]. Not all farmers adopt an agricultural innovation simultaneously, therefore it may be wise to accelerate the adoption pace of innovative early adopters to trigger wider and faster diffusion among less innovative farmers. For example, as part of Europe’s agro-ecological transition, finding ways to accelerate the dissemination of innovations to respond to environmental emergencies, including global warming, is crucial.

We tested for the impact of a nudge on the adoption process. Nudges are interventions that modify the decision context of individuals without altering their options or the structure of monetary incentives [5]. They are a cost-effective method aimed at guiding decisions towards desirable outcomes and they are increasingly considered as a policy option. A rising number of studies concern the effect of nudges on farmers’ decisions to adopt conservation practices or enroll in payment for environmental services schemes ([6–10], and see [11], for a review). Reported effect sizes are small, but authors insist that the benefit-cost ratio of nudging interventions remains advantageous, since these behavioral interventions are generally inexpensive to implement.

We focused on the role of farmer innovativeness, defined as a personality trait and potentially constitutive of a farmer’s self-identity [12]. Our nudge targeted the personality traits of farmers scoring high on the innovativeness scale, which is strongly associated to their self-image as “innovative farmers”. We chose to test a priming nudge defined as an unconscious activation of psychological factors such as norms, goals, or emotions. In this research, we addressed the following question: can the use of such nudge create a multiplier effect for accelerating adoption, or will the reactions of “less innovative” farmers backfire?

Our contribution is twofold. First, we designed and tested a measure of farmer innovativeness that is as reliable as possible. This latent variable is a behavioral trait that is not directly observable. We adapted the work of [13], which focuses on consumer purchasing decisions of innovative products, to take into account the specificity of decisions made by farmers in a professional context. Second, we experimentally tested the causal impact of a priming nudge designed to pique the interest of innovative farmers in the innovation presented to them using a randomized experiment embedded in a survey, from which we drew a control and a nudged group. We measured the effectiveness of this nudge on the stated ‘intention to adopt’ of targeted farmers (farmers with a high innovativeness score), as well as on the rest of the sample (farmers with a lower innovativeness score) to evaluate potential backfire effects.

We applied our analysis to the particular case of adoption of the “*Label bas carbone*” or “low-carbon label” (henceforth LCL) by farmers in France. The LCL is a certification framework for net carbon emissions abatement, which was established in 2018 as part of the toolbox mobilized by the French authorities to achieve the Green Deal’s 2050 carbon neutrality objective (European Commission, 2021). The LCL certifies voluntary farms that agree to adopt a set of agricultural practices resulting in proven avoided greenhouse gas emissions and/or additional carbon sequestration (in soil and/or trees). Certified carbon credits can then be sold by farmers on the voluntary market (Ministry of Environment, 2018) to firms that wish to offset their own emissions. Farmers can establish a kind of new “green business model”, as evoked by the European Commission in its new Carbon Farming/ Carbon Removal Regulation (CFCR Regulation 2024/3012) establishing a certification framework for permanent carbon removal.

We built an online survey for a large sample of French farmers, regardless of their type of agricultural production or socio-economic profile. The questionnaire consisted of four main parts: (1) farm’s characteristics; (2) agricultural practices on majority crops; (3) questions related to the LCL; and (4) respondent’s socio-demographic characteristics.

## 2 Materials and methods

In this section we detail how we defined the innovation adoption process (2.1), built a measure of farmer innovativeness (2.2), designed our nudge to leverage innovation adoption among (innovative) farmers (2.3), collected our data (2.4) and finally we present our statistical analysis (2.5).

### 2.1 Defining the innovation adoption process

We chose the LCL because it is an innovation that can be adapted to nearly all types of farming systems and that can be easily understood, while remaining relatively new and scarcely adopted. Nevertheless, to verify that respondents indeed consider LCL to be an innovation with attractive attributes, control questions were added to the survey about the LCL’s relative advantage, compatibility, complexity, feasibility, and observability.

Considering that LCL adoption is a process, we used a four-level proxy to characterize its adoption.

- **Y** = 1 if the farmer declared that he did not wish to obtain additional information on the LCL (later labelled: ***No information***);

- **Y** = 2 if the farmer declared that he wished to get more information but believed that the LCL is not of interest to his farm (later labelled: ***Want information***);

- **Y** = 3 if the farmer declared that the LCL is of interest to his farm but also stated that he did not intend to use it (later labelled: ***Interest***);

- **Y** = 4 if the farmer believed that the LCL is of interest to his farm and declared that he intended to adopt it (later labelled: ***Adopt***);

We also considered two additional levels indicating opposition to the LCL (**Y** = 0) and LCL already adopted (**Y** = 5).

### 2.2 Building a measure of farmer innovativeness

In our study, we used a measure of farmers’ “agriculture-specific innovativeness” to analyze their adoption of the LCL. However, as there is currently no measure of domain-specific innovativeness applicable to farmers available in the literature, we adapted the Goldsmith and Hofacker six-item scale [13], as shown in Table 1.

As this scale focused mostly on the speed of innovation adoption, [14] proposed complementing it with a dimension on information seeking, close to what [15] proposed. We therefore added two questions, also on a 5-item Likert scale:

*-“When adopting a new technology or agricultural equipment, I take my time to learn to master it properly”* and

*-“I do my best to make full use of all the features of new machinery and equipment or to take full advantage of a new farming practice”*.

**Table 1:** Psychometric scale used to measure farmer innovativeness (right column), adapted from [13] (left column).

Goldsmith and Hofacker (1991)^a,b^	Our psychometric scale^a,b^
**Compared to my friends** I own few rock albums.	**Compared to the neighboring farms,** the farming model on my farm is not very innovative.
In general, I am **the last in my circle of friends** to know the titles of the latest rock albums.	In general, **I am among the last farmers around me** to know what’s new in terms of farm equipment.
In general, **I am among the last in my circle of friends** to buy a new rock album when it appears.	In general, I am **among the last of the farmers around me** to adopt new farming practices.
If I heard that a new rock album was available in the store, **I would be interested enough** to buy it.	If I heard about new farming practices, **I would be interested enough** to test them.
l will buy a new rock album, **even if I haven’t heard it yet**.	I will adopt a new agricultural practice **even if I have never seen it applied before.**
I know the names of new rock acts **before other people do.**	I hear about new agricultural technologies **before other farmers do.**

^a^ *Likert scale for each item, ranging from “1 - Strongly disagree” to “5 - Yes, strongly agree”*

^b^ *We use bold font to emphasize how we have adapted the scale to the agriculture domain. (Bold font was not used in surveys sent to respondents).*

Finally, to ensure the reliability of the farmer-adapted scale, as in [13], we added the following three control questions to measure item validity of innovativeness:

*-“How often do you keep up-to-date with the latest agricultural news?”* ***Keepup_Latest_News*** variable coded from 1 (Never) to 4 (Often).

-*“How often do you talk to an agricultural advisor, cooperative members, or other farmers about innovations in your sector?”* ***Often_Exchang*e** variable coded from 1 (Never) to 4 (Often)

-*“Do you appreciate these moments of information-gathering and exchanges on innovations in agricultural practices?”* ***Like_Exchange*** variable coded from 1 (No, I do not like it at all) to 5 (Yes, I appreciate it very much).

The scores for each question are added together and lead to an innovativeness aggregated score per individual (***PSYCHO_INNOV*** variable for PSYCHOmetric scale to measure INNOVativeness) ranging from 8 to 40. We followed the approach of [16] (a study on risk aversion) and added a single question as a proxy for innovativeness (***SR_INNOV*** for Self-Reported INNOVativeness): *“How willing are you to innovate on your farm?”* (1: Not willing at all to 10: Very willing).

We tested different ways to classify our respondents in the “more innovative” or “less innovative” categories. The classification used to present our results combined the two measures of innovativeness. First, we identified the 20% of farmers who have the highest score using each of the two measures. Second, classifications from the two measures were compared to assess consistency. To minimize potential misclassification, respondents whose classifications differed across the measures were excluded from subsequent analyses. Third, using a conservative approach, we defined the Innovativeness (***INNOV)*** dummy variable as 1 if the respondent is classified as “more innovative” by both measures and 0 if the respondent is classified as “less innovative” by both measures.

### 2.3 Designing a nudge to leverage innovation adoption among farmers

We used two descriptions of the LCL randomly allocated to farmers in the survey. One was a benchmark description of the LCL that outlined the different steps a farmer needs to take to become LCL-certified. The other was the same LCL description, providing exactly the same information but using a lexical field associated with innovation (e.g., “innovativeness”, “inventiveness”, “experimenting”, “development”, “be an example for others”, “be the first”…) in order to trigger adoption among farmers with a high innovativeness score. Table 2 presents the two versions of the LCL description.

Farmers vary in their attitudes and preferences toward innovation, and thus may respond differently to our priming nudge depending on their innovativeness score. We therefore tested the following hypothesis with our randomized experiment:

***H1*** *: The impact of the priming nudge on the more innovative farmers’ intentions to adopt the LCL is positive.*

We also test for a potential backfire effect:

***H2*** *: The impact of the priming nudge on less innovative farmers’ intentions to adopt the LCL is null or even negative.*

**Table 2:** LCL description in the questionnaire (two versions).

Benchmark version – control group^a^	Nudged version – treatment group^a^
The LCL, created in 2019, allows farmers to be paid for the services they provide to mitigating climate change.	The LCL, created in 2019, is the **first carbon standard in agriculture**. It allows farmers **who wish to evolve and experiment** to be paid for the services they provide to mitigating climate change.
The steps of the certification process are:	The steps of the certification process are:
1) Carry-out a “carbon footprint” analysis of farm activities. An approved certification organization carries out this analysis and, with the farmer, identifies the progress that can be made to reduce emissions and/or store more carbon.	1) Carry-out a “carbon footprint” analysis of farm activities. An approved certification organization carries out this analysis and, with the farmer **who wants to renew his practices**, identifies the progress that can be made to reduce emissions and/or store more carbon.
2) Take actions to improve the carbon balance. Reducing greenhouse gas emissions and/or increasing carbon storage will imply, for example, a change in livestock feed, fertilization or tillage, or planting trees, etc.	2) Take **innovative** actions to improve the carbon balance. **Renew, test solutions, be inventive!** Reducing greenhouse gas emissions and/or increase carbon storage will imply, for example, a change in livestock feed, fertilization or tillage, or planting trees, etc.
3) Certify the improvements made to the initial carbon balance. Net emission reductions are officially certified and registered in a dedicated register.	3) Certify the improvements made to the initial carbon balance. Net emission reductions are officially certified and registered in a dedicated register.
4) Obtain payments for environmental services. Private companies or communities can finance these certified reductions at a price negotiated on a case-by-case basis.	4) Obtain payments for environmental services. Private companies or communities can finance these certified reductions at a price negotiated on a case-by-case basis.
Why commit? -Benefit from additional income; -Have the satisfaction of contributing to the environment.	Why commit? -Benefit from additional income; -**Renew farming practices** and have the satisfaction of **seeing the agricultural sector** contribute to the environment; -**Be a reference for other farmers and help them to commit as well.**

^a^ *We use bold font to emphasize how we adapted the LCL description. (Bold font was not used for the survey provided to respondents.)*

### 2.4 Data collection

The distribution of the survey was outsourced to the polling institute BVA Group, which complies with General Data Protection Regulation (GDPR) policies and abides by the ICC/ESOMAR International Code on Market and Social Research regarding ethics in social science research. The survey was conducted online, in accordance with GDPR guidelines. Consent was informed and written approval was gathered by BVA. In accordance with INRAE’s ethical guidelines, we also conducted a self-assessment of the potential ethical risks associated with our survey. After consulting with the ethics officer of our research unit, we received a confirmation that formal approval from an institutional review board (IRB) was not required. The survey was conducted with Limesurvey software and sent by BVA to its entire panel of 90,000 French farmers (after an initial test on a small sample of farmers). The email invited farmers to respond to a survey conducted by researchers on the remuneration of services provided by agriculture and was written to encourage as many farmers as possible to click on the survey link. In addition, the email guaranteed that the survey was anonymous and specified that it would take about 15 minutes to complete. The first wave was launched on June 15, 2022. We collected 2,500 complete responses. Because early summer is usually a busy time for farmers, we decided to postpone the re-launch of the survey until winter. The second wave was launched on December 7th, 2022, and ended on January 18, 2023, enabling us to collect 3,505 additional responses, for a total of 6,005 complete responses. Of the 90,000 farmer email addresses in the BVA panel, several were invalid, and many emails were not read. Since we do not know how many farmers read our invitation, we cannot compute a response rate. Nevertheless, we know that there were 14,534 clicks on the survey link, and that 41% of them resulted in a complete questionnaire, which shows the level of interest of farmers in completing our survey.

### 2.5 Statistical analyses

We first examined the correlations between the self-reported innovativeness (***SR_INNOV***), the psychometric scale to measure innovativeness (***PSYCHO_INNOV***), and the control variables capturing information-seeking and engagement with peers. Then, we constructed a contingency table between the two innovativeness measures to derive the binary variable ***INNOV***, which identified respondents as either “more innovative” and “less innovative”. To assess the relevance of the innovativeness proxy we used, we described the distribution of the average innovativeness rate according to the different modalities of Y (intention to adopt LCL). Additionality, we explored the behavioral and contextual determinants of innovativeness using a logistic regression to identify behavioral factors associated with being classified as “more innovative”.

Before measuring the impact of the nudge, we examined our sample descriptively and conducted balancing tests to ensure the comparability between treatment and control groups. The impact of the priming nudge on farmers’ intentions to adopt the LCL (Y, on a 1–4 scale) was assessed by comparing mean outcomes between the control and treatment groups using Student t-tests, complemented by effect size estimates using Cohen’s d. To account for potential heterogeneity in responses, we considered the possibility of a backfire effect of the nudge on less innovative farmers, as hypothesized in H2, whereby a negative effect on this group could offset any positive effect on the more innovative farmers. Separate analyses were therefore conducted for the “more innovative” and “less innovative” groups, allowing us to test both the intended effect and the potential backfire. We conducted retrospective power analyses to test if our sample size was sufficient to detect the smallest effects of interest with a high level of statistical confidence. Additionality, we performed two robustness analyses. We examined binary adoption outcomes (ADOPT = 1 if Y = 4; ADOPT = 0 if Y = 1–3, excluding Y = 0 or 5). We also explored the behavioral and contextual determinants of LCL adoption using ordered logit regressions that included the treatment variable (exposure to the nudge), innovativeness and perceptions of the LCL’s attributes.

## 3 Results

### 3.1 Sample description

Table 3 presents the main socio-demographic characteristics of our sample. A large majority of our respondents were male (87%). Respondents were slightly younger than the average farmer in France. Those over 60 were mostly under-represented in favor of those under 55, which is common in online surveys. Our sample was also composed of large farms, to the detriment of small farms of less than 50 hectares, especially those less than 20 hectares. Table 3 shows that our sample was not representative of the French farmers.

**Table 3:** Sample characteristics.

	Our sample	France (data 2016)
**Mean UAA**	155 ha	69 ha
**Organic farming**	11.9%	12.1%
**Average age**	48.6 years	52.2 years
**Education (frequency)**		
*No diploma*	*0.8%*	*6%*
*Lower than high school diploma*	*12.4%*	*42%*
*High school diploma and undergraduate*	*63.4%*	*28%*
*Graduate studies and above*	*23.4%*	*24%*
**Main activity (frequency)**		**France (data 2020)**
*Field crops*	*44.6%*	*28.69%*
*Market gardening and horticulture*	*1.2%*	*3.94%*
*Wine growing/Viticulture*	*2.4%*	*15.14%*
*Fruits & other permanent crops*	*1.1%*	*3.92%*
*Dairy cattle*	*13.4%*	*8.99%*
*Meat cattle*	*8.5%*	*12.42%*
*Mixed cattle*	*0.5%*	*2.14%*
*Sheep, goats*	*4.1%*	*9.10%*
*Pigs and poultry*	*2.6%*	*4.79%*
*Mixed crops – mixed livestock*	*20.8%*	*10.41%*
*Unclassified farms*	*0.8%*	*0.47%*
**Total number of farms**	**6,005 farms**	**389,779 farms**

The various sample characteristics were well balanced between the control group (3,035 responses, 50.5%) and the treatment (nudged) group (2,970 responses, 49.5%). We found no significant difference between the two groups in terms of gender, age, utilized agricultural area, type of farming, respondent marital status, or education level.

**Fig 1:**
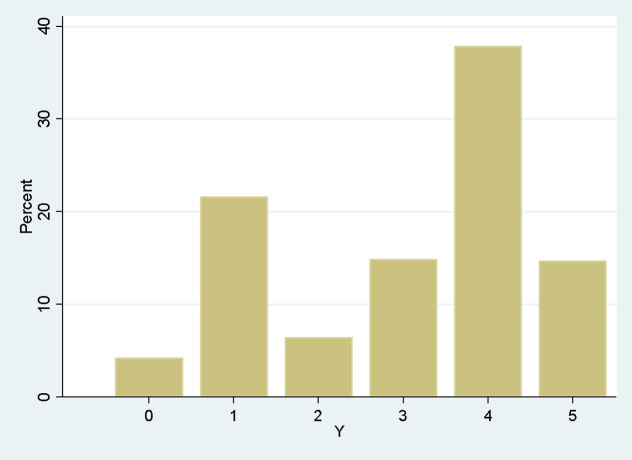
Distribution of the discrete variable of interest Y: Adoption of the LCL.

Table 4 and Fig 1 show the distribution of respondents according to Y.

**Table 4:** Distribution of the discrete variable of interest Y: Adoption of LCL^a.^

Y: Adoption of LCL		Number and share of respondents
***Opposed*** (**Y** = 0)	Opposed to the LCL	257 (4.28%)
***No information*** (**Y** = 1)	Does not want information on the LCL	1,299 (21.65%)
***Wants information*** (**Y** = 2)	Would like to have information on the LCL	390 (6.50%)
***Interest*** (**Y** = 3)	Believes that the LCL could be of interest to his farm but not ready to commit	894 (14.90%)
***Adopt*** (**Y** = 4)	Wants to get involved in the LCL	2,275 (37.92%)
***Already adopted*** (**Y** = 5)	Already involved in the LCL	884 (14.74%)

^*a*^ *Six observations are missing (three from the control group and three from the treatment group). These missing values are linked to a technology glitch following an internet connection problem.*

Almost 15% of respondents declared that they “have already started the LCL certification process within their farm” and 38% “wish to apply for LCL certification on their farm”. Only 4% indicated that they were opposed to the LCL, and 22% were not interested in the LCL, i.e., did not want information about it.

### 3.2 Farmer innovativeness

**Fig 2:**
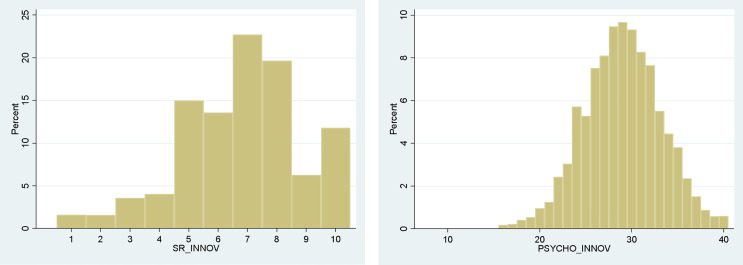
Distribution of our two measures: self-reported innovativeness (left panel) and psychometric scale to measure innovativeness (right panel).

The mean of the self-reported measure of innovativeness (***SR_INNOV)*** over the total sample was 6.80, with scores ranging from 1 to 10. The second measure, the psychometric scale to measure innovativeness (***PSYCHO_INNOV***) was the combination of the adapted Goldsmith and Hofacker (1991) scale [13] and Du et al. (2021)’s two questions [14], adapted to the agricultural context. The average score for the psychometric scale (***PSYCHO_INNOV)*** was 28.96, with scores ranging from 8 to 40. The distribution of each measure was presented in Fig 2.

Table 5 shows that the two measures were valid, as they were highly correlated with each other and both positively correlated with the three control questions. The psychometric scale (***PSYCHO_INNOV***) appeared to perform somewhat better than the self-reported innovativeness (***SR_INNOV***), as it was more correlated with the two control variables ***Keepup_Latest_News*** and ***Often_Exchange***.

**Table 5:** Correlations of scales for measuring innovativeness with control variables.

	*SR_INNOV* Self-reported innovativeness	*PSYCHO_INNOV* Psychometric scale for innovativeness
***SR_INNOV*** Self-reported innovativeness	1	
***PSYCHO_INNOV*** Psychometric scale	0.5876	1
***Keepup_Latest_News*** Keeps up-to-date with latest agricultural news (1 never to 4 often)	0.2833	0.3564
***Often_Exchange*** Talks about innovation in own sector (1 never to 4 often)	0.2847	0.3256
***Like_Exchange*** Appreciates information gathering and exchanges on innovations (1 do not like it at all to 5 appreciate it very much)	0.3792	0.3634

Table 6 aimed to code the binary variable ***INNOV***. Farmers were classified as “more innovative” if they ranked in the top 20% on both measures – score higher or equal to nine (9) for ***SR_INNOV*** (18%) and higher or equal to 33 for ***PSYCHO_INNOV*** (20%) – and “less innovative” if they ranked below this threshold on both. We found that 4,313 farmers (72%) and 580 (10%) were identified as “less innovative” and “more innovative” by the two measures, respectively. The 1,112 farmers (18%) classified differently depending on the measure considered were eliminated from the rest of the analysis to minimize misclassification. This reduced our sample size to 4,893 observations for the ***INNOV*** binary variable.

**Table 6:** Table of contingencies between the two innovativeness measures.

		*PSYCHO_INNOV* Psychometric scale for innovativeness		Total
		Less innovative	More innovative	
***SR_INNOV*** Self-reported innovativeness	Less innovative	4,313 (72%)	605 (10%)	4,918 (82%)
	More innovative	507 (8%)	580 (10%)	1,087 (18%)
Total		4,820 (80%)	1,185 (20%)	6,005 (100%)

Table 7 shows the distribution of ***INNOV*** as a function of LCL adoption. Excluding respondents who declared they are opposed to the LCL (**Y** = 0), we showed that the proportion of more innovative farmers (average rate of ***INNOV***) increased with the value of **Y**, i.e., the scaled intention to adopt LCL. A Student test also showed that the mean of **Y** was significantly higher (one-sided test: 0.000) for more innovative farmers (3.66) than for less innovative farmers (2.90). The most innovative farmers were more likely to score higher in the intention to adopt the LCL than are less innovative farmers.

**Table 7:** Average rate of innovativeness according to Y.

Y: Adoption of LCL	Average rate of *INNOV*
***Opposed*** (**Y** = 0)	12%
***No information*** (**Y** = 1)	5%
***Wants information*** (**Y** = 2)	6%
***Interest*** (**Y** = 3)	6%
***Adopt*** (**Y** = 4)	15%
***Already adopted*** (**Y** = 5)	24%

Supplementary analyses about the behavioral and contextual determinants of innovativeness are reported in Appendix A in S1 Appendices.

### 3.3 Nudge impact assessment

*Balancing tests:* Table 8 shows that our control and treatment groups were well balanced on the several measures of respondents’ innovativeness. Any difference between the two groups’ intentions to adopt the LCL could therefore be attributed to the nudge only.

**Table 8:** Average innovativeness scores, by type of innovativeness, in the control and treatment groups.

Variable	Whole sample	Control group	Nudged group	Mean comparison test: control vs. nudge p-value
***SR_INNOV*** Self-reported innovativeness (scale 1–10)	6.80 (2.03)^a^	6.77 (2.04)	6.83 (2.03)	0.2457
***PSYCHO_INNOV*** Psychometric scale for innovativeness (scale 8–40)	28.96 (4.23)	28.93 (4.27)	28.98 (4.20)	0.6601
***INNOV*** (dummy)	0.1185 (0.3233)	0.1181 (0.3228)	0.1189 (0.3238)	0.9316

^*a*^ *Standard deviation*

Excluding the 884 farmers already committed to the LCL (**Y** = 5) and the 257 opposed to the LCL (**Y** = 0), we still found that our two groups were still well balanced on that sub-sample of 4,858 observations.

Table 9 further shows that the distribution of farmers across the **four-level adoption variable Y** values was not significantly different between the control and treatment groups.

**Table 9:** Number of farmers, by level of adoption, in the control and treatment groups.

Y: Adoption of LCL	Control group	Nudged group	Total
***No information*** (1)	621	678	1,299
***Wants information*** (2)	185	205	390
***Interest*** (3)	459	435	894
***Adopt*** (4)	1,144	1,131	2,275
**Total**	2,409	2,449	4,858

*Average treatment effect*: On average, as shown by column **Total** of Table 10, farmers who were nudged (mean of **Y** = 2.82, SD 1.27) were not more willing to adopt the LCL than farmers in the control group (mean of **Y** = 2.88, SD 1.25). The average impact of the nudge on our total sample was not significantly different from 0. In addition, we calculated Cohen’s d (mean difference between the two independent groups divided by the pooled standard deviation). We obtained a very low Cohen’s d (0.048).

**Table 10:** Mean (and SD) of the four-level adoption variable Y for control and treatment groups, by category of innovativeness.

	Total (4,858 farmers)	Less innovative (3,605 farmers)	More innovative (391 farmers)
**Control group (50%)**	2.88 (1.25)	2.77 (1.27)	3.43 (1.02)
**Nudged group** **(50%)**	2.82 (1.27)	2.71 (1.28)	3.25 (1.16)
**Total**	2.85 (1.26)	2.74 (1.27)	3.34 (1.09)
Student test (two-sided test)^a^	0.1091	0.2151	0.1120
**Cohen’s d** ^b^	−0.048	−0.047	−0.165

^a^None of the differences is significant at 1% (***), 5% (**) or 10% (*)

^b^Cohen’s d calculated as the mean difference between the two groups divided by the pooled standard deviation.

The last two columns of Table 10 measured the impact of the nudge by distinguishing its impact on two classes of farmers: the less innovative and the more innovative farmers. Results were the same for both.

*Power analysis:* We conducted a retrospective power analysis to measure the minimum detectable effect attainable with our sample sizes. For a statistical power of 0.8, and a significance level of 0.05, and given measured standard deviations:

-The minimum detectable effect size on **Y** of the nudge in the sub-sample of 391 “more innovative” farmers was 0.31 (relative effect of 9.04%). Table 10 shows that we measured an absolute treatment effect of −0.18 (calculated as 3.43–3.25), equivalent to a relative treatment effect of −5.25%. If this effect existed, detecting it with a significance level of 5% would require at least 1,200 observations (600 farmers in treated and control groups, respectively).

-The minimum detectable effect on **Y** of the nudge in the sub-sample of 3,605 “less innovative” farmers was 0.12 (4.29%), whereas the measured effect was only −0.06 (−2.12%). To test whether this treatment effect was truly significant at 5% would require at least 18,400 observations (9,200 farmers in treated and control groups, respectively).

*Supplementary analyses:* Robustness checks yielded consistent results. Appendix B in S1 Appendices shows that the null effect of the nudge was unchanged when using a binary variable instead of the four-level adoption variable **Y**. Additional models separating the effects of the priming nudge from farmers’ pre-existing attitudes and contextual factors were reported in Appendix C in S1 Appendices (four-level adoption variable **Y**) and Appendix D in S1 Appendices (binary adoption variable). They provided a comprehensive understanding of the drivers of LCL adoption.

## 4 Discussion

*Farmers’ innovativeness:* Our study contributes to the literature, in general and in economics, on farmer innovativeness by adapting a psychometric scale to the agricultural domain. In the behavioural science and in the marketing science literature, the concept of innovativeness was initially approached through a single psychological trait characterizing the individual with a kind of general or innate innovativeness that would weigh in their decisions, regardless of the type of innovation. Yet [13] developed the concept of domain-specific innovativeness. Indeed, “being innovative” does not imply being innovative in all domains—an individual may be open to new foods but reluctant to use new technologies, for example. Prior research has shown that using an inappropriate innovativeness scale can lead to erroneous results [17].

Innovativeness has received little attention in the economics literature. Economists typically measure innovativeness using proxy indicators, such as the type and number of new practices adopted by farmers [18–19] or their past use of new technologies [20] but not using psychometric scales let alone a domain-specific psychometric scale. In this article, we assumed that innovativeness in the agriculture domain should not be elicited at an overall level, but rather at a specific level, i.e., that of the professional environment in which the farmer has an expert view on innovations. We adapted the Goldsmith and Hofacker six-item scale [13] that measures the ability to innovate in a specific domain among *consumers*. It is a balanced scale with both positive and negative items, which has proven to be very reliable, with strong predictive validity [21]. As such, this scale has been deemed transferable to several other domains: [22] use it in the field of technology and [23] in the field of fashion. [24] use it in several countries (Canada, Israel, France), and [25] offer a meta-analysis of the scale based on 78 consumer studies.

We compared this psychometric scale of innovativeness tailored to the agricultural context with a one-item self-reported innovativeness assessment. As expected, the two measures were strongly correlated, and both showed significant positive correlations with the three control variables, supporting their validity. Importantly, farmers scoring higher on either measure were more likely to adopt innovations, confirming the predictive validity of these tools.

*No nudge effect:* Economists have used priming as an instrumental tool, for example, to increase donations to not-for-profit organizations ([26]) or to activate self-identity ([27]; [28]; [29]). However, the literature shows that poorly targeted nudges—especially those relying on priming-like mechanisms—may backfire and produce effects opposite to those intended. For example, [30] document a backfiring effect of a salience nudge aimed at curbing consumer overspending by highlighting opportunity costs, the nudge reduced spending among “tightwads” but increased it among “spendthrifts,” resulting in lower overall welfare. This evidence shows that “one-size-fits-all” nudges can backfire when they target a single attitude or trait within a heterogeneous population. Similar conclusions are reached in other contexts ([31]). In a large-scale RCT, [29] find that a self-identity priming nudge has no effect on household energy conservation unless it is targeted to individuals with past pro-environmental behavior and high energy use. In the agricultural domain, [32] show that while nudges can increase compliance with environmental regulations, a social comparison nudge may also exacerbate non-compliance among a deviant sub-population, potentially due to “reactance”, which refers to the defiance or negative reaction triggered by a signal perceived as not legitimate. This body of evidence suggests that similar mechanisms may limit the effectiveness of our priming nudge. Accordingly, we examine heterogeneous effects across farmers with different levels of innovativeness.

Our main result was that the priming nudge, designed to encourage the most innovative farmers to adopt the proposed innovation, had no significant effect on their stated intention to do so (Hypothesis 1 not confirmed). The nudge also had no counteracting effect on the group of less innovative farmers (Hypothesis 2 confirmed). Our study was conducted with sufficient statistical power to provide robust results. Our power analysis (very low Cohen’s d [33]) allowed us to safely conclude that the treatment effects measured through our experiment was either not significantly different from 0 or was very low, at 5% or below.

To achieve this level of statistical power, it was necessary to recruit a large number of respondents. To this end, we ensured that our survey was relevant to all farmers, regardless of their type of agricultural production or socio-economic profile. This consideration also guided our choice in favor of the low-carbon label as the innovation under study. In addition, because participation in the survey was voluntary and anonymous, the questionnaire was deliberately kept short and clear in order to maximize response rates. Although we avoided terms related to innovation and the environment in the invitation letter to limit potential respondent selection bias, our sample was not representative of the population of French farmers. However, no conclusion could be drawn from the fact that certain categories of farmers are over- or under-represented in our sample, because the panel of farmers who received the invitation was itself not representative. Importantly, the fact that our sample was not representative was not problematic as such, since our goal was to understand the behavioral determinants of innovation adoption among farmers and to test the impact of our nudge using a control group.

Additional exploratory analyses examined potential heterogeneity by farm scale, using a proxy based on utilized agricultural area. Large-scale farmers appear more engaged in the LCL and slightly more innovative than small-scale farmers. However, the priming nudge remains largely ineffective across both groups.

The null impact of our innovation-framed priming nudge should not be interpreted as evidence against behavioral interventions more broadly, nor against priming strategies in other settings. However, it leads us to question the effectiveness of nudges in professional context, where financial stakes and risks of innovation adoption are high and where decision-makers tend to deliberate carefully before making a decision.

Drawing on numerous examples, [34] showed that nudges are sometimes ineffective, or less effective than expected, and in some cases counterproductive, leading to unintended consequences. A large body of literature in psychology has sought to understand the mechanisms through which priming exerts (or fails to exert) significant effects (see [35], for a review). A quantitative review by [36] analysed the effect sizes of nudges across various domains and found a substantial heterogeneity across nudge types and studies, with some effects being null and even negative. [37] reach the same conclusions with a meta-analysis and [38] confirmed that, due to publication bias, published effect sizes of nudges are often much larger than observed effects.

None of the analyses mentioned above focused on the agriculture domain. As the study by [36] shows, most of the empirical evidence described in the scientific literature on nudge effects concern the behaviors of patients, consumers, or citizens, typically involving low-stakes, short-term decisions. By contrast, longer-term, higher-stakes decisions taken in a professional context may be less susceptible to this type of innovation-framed priming intervention. This is consistent with the idea that nudges primarily target the automatic, intuitive, and thinking process of System 1 rather than the more conscious, rational, and logical thinking of System 2 [39]. Yet this question remains under-explored and is rarely mentioned in studies testing nudges on farmers.

Empirical evidence from agriculture supports this observation. For instance, large-scale randomized controlled trials (RCT) conducted in France and the United States reported modest or null impacts of nudges, such as peer comparisons and social norm messages [6,8,9]. [40] reported that a salience nudge emphasizing local biodiversity benefits was ineffective, while [9] found that injunctive norm and social signaling nudges did not significantly influence farmers’ adoption of a simple innovation such as a mobile app.

Our study provided new evidence that an innovation-framed priming nudge targeting farmers may have no detectable effect on stated adoption intentions, even with a very large sample.

## 5 Conclusion

This article adapted established methods from marketing science to assess farmers’ innovativeness. We examined whether innovativeness was positively correlated with the adoption of a new innovation and tested the effect of a priming nudge designed to increase adoption by specifically targeting innovative farmers. We showed that farmers can be classified according to their level of innovativeness using both a self-reported measure and a psychometric scale adapted to the agricultural context, and that farmers who score higher on these innovativeness scales were more likely to adopt new innovations. However, our results indicated that the innovation-framed priming nudge had no significant effect on adoption rates, regardless of farmers’ innovativeness levels. It neither increased the stated adoption intentions of the most innovative farmers nor produced any negative backfire effects by discouraging less innovative farmers. While we cannot definitively determine whether the lack of effect is due to the subtlety of the intervention, we recognize that this is a plausible explanation.

It is worth mentioning the recent article by [41]. They contrast two types of interventions: interventions that focus solely on changing individual behavior through behavioral approaches (i-frame policies, such as nudges); and policies that change the system of rules, norms and institutions (s-frame policies, such as taxes or regulations). They note that the last decade has seen growing enthusiasm for i–frame policies although recent evidence show that they only have a very modest effect. They argue that the interest of research and governments for i-frame interventions may have deflected attention and support away from s-frame policies, sometimes slowing down the pace of necessary systemic reforms. Our findings added to this literature by suggesting that nudging policies, and specifically innovation-framed priming nudge, alonemay not suffice to change farmers’ decisions.

## Supporting information

S1 Appendices This file contains: **Appendix A.** Logistic regression of innovativeness on behavioral factors. **Appendix B.** Effect of the nudge using the ADOPT binary variable. **Appendix C.** Behavioral and contextual factors of LCL adoption using the four-level adoption variable Y. **Appendix D.** Behavioral and contextual factors of LCL adoption: logistic regression of ADOPT dummy variable.
(PDF)

S2 Data This file contains the data of the study. (DTA)
