# Supplementary material for: A priming nudge targeting innovative farmers: A large-scale survey experiment
Source: PLoS One. 2026 Mar 26;21(3):e0345658. doi: 10.1371/journal.pone.0345658 (PMC13020822; doi:10.1371/journal.pone.0345658)
Supplement: S1 Appendices — Appendix A. Logistic regression of innovativeness on behavioral factors. Appendix B. Effect of the nudge using the ADOPT binary variable. Appendix C. Behavioral and contextual factors of LCL adoption using the four-level adoption variable Y. Appendix D. Behavioral and contextual factors of LCL adoption: logistic regression of ADOPT dummy variable. (PDF) [file pone.0345658.s001.pdf]

Appendix A. Logistic regression of innovativeness on behavioral factors

Table A.1 shows first that the probability of belonging to the most innovative group was higher when individuals were more willing to take risks and have a strong preference for the future, favoring future profitability over present profitability. Having higher environmental preferences also increased the probability of belonging to the most innovative group. However, being sensitive to social norms and being willing to adapt to new regulations both reduced the probability of belonging to the most innovative group.

Table A.1. Logistic regression of innovativeness on behavioral factors

**Independent variable**	**Description**	**Coef. (Std. Err.)**
Risk preference	Willingness to take risks (1 to 10)	0.717*** (0.035)
Time preference	Willingness to give up a profitable investment today in order to invest in better technologies in the future (1 to 10)	0.193*** (0.029)
Other farmers' adoption of LCL	Inclination to adopt the LCL if other farmers did the same: low (1) to high (5)	-0.357*** (0.051)
Adaptation	Adaptation to a new regulation which would require a costly adaptation: low (0), medium (1), high (2)	-0.169** (0.068)
Environmental preferences	Five-item scale: score from 0 to 20	0.122*** (0.017)
Constant		-8.40*** (0.361)
**#Obs**	4,893	
**Log likelihood and test**	-1,224.64***	

Appendix B. Effect of the nudge using the ADOPT binary variable

Table B.1 shows that the results were unchanged if we consider the binary ***ADOPT*** variable defined as ***ADOPT*** = 1 if **Y** = 4, ***ADOPT*** = 0 if **Y** = 1, 2 or 3 [***ADOPT*** = . (missing value) if **Y** = 0 or 5].

Table B.1. Mean (and SD) of binary adoption (*ADOPT*) for control and treatment groups, by category of innovativeness

	**Total** (4,858 farmers)	**Less innovative** (3,605 farmers)	**More innovative** (391 farmers)
**Control group**	0.4749	0.4273	0.7077
	(0.4995)	(0.4948)	(0.4560)
**Nudged group**	0.4618	0.4099	0.6480
	(0.4986)	(0.4919)	(0.4788)
**Total**	0.4683	0.4186	0.6777
	(0.4990)	(0.4934)	(0.4679)
Student test (two-sided test)[a]	0.3617	0.2889	0.2073

[a] None of the differences is significant at 1% (***), 5% (**) or 10% (*)

Furthermore, we checked that the reason why the nudge had no significant effect on the most innovative farmers was not related to the fact that these farmers would not see the LCL as an innovation. To verify this, we compared the effect of the nudge on the sub-group of the most innovative farmers, who considered the LCL to be an innovation. Table B.2 shows that we found no significant difference in the willingness to adopt the LCL and no evidence that considering LCL to be an innovation affected the impact of the nudge.

Table B.2. Mean (and SD) of binary adoption (*ADOPT*) for control and treatment groups, if farmers consider LCL as an innovation and are among the most innovative farmers

	ADOPT ***Mean (SD)***
**Control group** (134 farmers)	0.8209 (0.3849)
**Nudged group** (123 farmers)	0.7886 (0.4100)
**Total** (257 farmers)	0.8054 (0.3966)
Student test (two-sided test)[a]	0.5156

[a] None of the differences is significant at 1% (***), 5% (**) or 10% (*)

Appendix C. Behavioral and contextual factors of LCL adoption using the four-level adoption variable Y

In Table C.1, we were interested in identifying the behavioral and contextual factors that can explain the declared intention of farmers in our sample to adopt the LCL.

Table C.1. Ordered logit regression of the four-level adoption variable Y

Independent variables	Variable description	Coef. (Std. Err.)[a]
Nudge	Dummy for control (0) vs nudged group (1)	-0.103 (0.065)
INNOV	Dummy for less (0) vs more (1) innovative farmers	0.381*** (0.143)
Risk preferences	Willingness to take risks (1 to 10)	0.023 (0.019)
Time preferences	Willingness to give up a profitable investment today in order to invest in better technologies in the future (1 to 10)	-0.001 (0.019)
Favorable context	Considering the context favorable for adopting the LCL (1 to 10)	0.408*** (0.021)
Other farmers' adoption of LCL	Inclination to adopt the LCL if other farmers did the same: low (1) to high (5)	0.135*** (0.037)
Adaptation	Adaptation to a new regulation which would entail a costly adaptation: low (0), medium (1), high (2)	-0.019 (0.043)
Environmental preferences	Five-item scale: score from 0 to 20	0.046*** (0.011)
LCL known	The farmer knew about the LCL before the survey, No (0), Yes (1)	0.254*** (0.072)
LCL innovative	The LCL is an innovative solution. No, not at all (0) to Yes, absolutely (4)	0.361*** (0.048)
LCL better option	The LCL is a better option for reducing farm's impact on the climate than more traditional schemes such as agri-environmental measures. No, not at all (0) to Yes, absolutely (4)	0.259*** (0.045)
LCL complex	The LCL is complex to understand and mobilize. No, not at all (0) to Yes, absolutely (4)	0.071* (0.040)
LCL compatible	The LCL is compatible with farm's objectives (in terms of needs, constraints, and values...) No, not at all (0) to Yes, absolutely (4)	0.530*** (0.048)
LCL observable	It is possible to observe the implementation of the LCL among farming neighbours or in professional circle. No, not at all (0) to Yes, absolutely (4)	-0.141*** (0.040)
LCL expe	Before committing to a LCL, it is possible to try it out with the help of advisors. No, not at all (0) to Yes, absolutely (4)	0.413*** (0.042)
**/cut1**		5.279 (0.244)
**/cut2**		5.869 (0.248)
**/cut3**		7.050 (0.239)
**#Obs.**	3,996	
**Log likelihood and test**	-3970.268***	

[a] Significance levels at 1% (***), 5% (**) or 10% (*)

As already shown, innovativeness (***INNOV***) increased the four-level adoption variable Y. Having strong environmental preferences and perceiving the context as favorable to adopting the LCL also increased the likelihood of adoption. Moreover, the finding that adoption probability rose when other farmers were also adopting the LCL suggests that farmers were influenced by descriptive social norms. To ensure the robustness of these results, control variables related to the innovation's attributes were included, so that the estimated effect of the treatment (the nudge) was not biased by farmers' prior perceptions of the LCL and its characteristics. As expected, most of the LCL's "positive" attributes (***LCL innovative***, ***LCL better option***, ***LCL compatible***, ***LCL expe***) had a positive impact on adoption. However, ***LCL observable*** displayed a significant negative impact, which was both surprising and intriguing. This result suggests that farmers who had directly observed the implementation of the LCL on their neighbors' farms were less inclined to adopt it themselves. In contrast, farmers who only knew that other, unfamiliar farmers had adopted the LCL as indicated in the "***Other farmers' adoption of LCL***" variable, tended to show stronger intentions to adopt. Together, these two results suggested that farmers who had implemented the LCL on their farm may not have had a positive experience and may have shared their dissatisfaction with their peers.

Appendix D. Behavioral and contextual factors of LCL adoption: logistic regression of ADOPT dummy variable

Table D.1. Logistic regression of ADOPT

**Independent variables**	**Variable description**	**Coef. (Std. Err.)[a]**
Nudge	Dummy for control (0) vs nudged group (1)	-0.084 (0.077)
INNOV	Dummy for less (0) vs more (1) innovative farmers	0.286* (0.157)
Risk preferences	Willingness to take risks (1 to 10)	0.041* (0.023)
Time preferences	Willingness to give up a profitable investment today in order to invest in better technologies in the future (1 to 10)	0.019 (0.023)
Favorable context	Considering the context favorable for adopting the LCL (1 to 10)	0.401*** (0.025)
Other farmers' adoption of LCL	Inclination to adopt the LCL if other farmers did the same: low (1) to high (5)	0.018 (0.043)
Adaptation	Adaptation to a new regulation which would entail a costly adaptation: low (0), medium (1), high (2)	-0.036 (0.051)
Environmental preferences	Five-item scale: score from 0 to 20	0.032** (0.013)
LCL known	The farmer knew about the LCL before the survey, No (0), Yes (1)	0.115 (0.085)
LCL innovative	The LCL is an innovative solution. No, not at all (0) to Yes, absolutely (5)	0.289*** (0.056)
LCL better option	The LCL is a better option for reducing farm's impact on the climate than more traditional schemes such as agri-environmental measures. No, not at all (1) to Yes, absolutely (5)	0.276*** (0.053)
LCL complex	The LCL is complex to understand and mobilize. No, not at all (1) to Yes, absolutely (5)	0.017 (0.047)
LCL compatible	The LCL is compatible with farm's objectives (in terms of needs, constraints, and values...) No, not at all (1) to Yes, absolutely (5)	0.506*** (0.056)
LCL observable	It is possible to observe the implementation of the LCL among farming neighbors or in professional circle. No, not at all (1) to Yes, absolutely (5)	-0.073 (0.046)
LCL expe	Before committing to the LCL, it is possible to try it out with the help of advisors. No, not at all (1) to Yes, absolutely (5)	0.391*** (0.050)
Constant		-6.324*** (0.302)
**#Obs.**	3,996	
**Log likelihood and test**	-2038.5856***	

[a] *Significance levels: 1% (***), 5% (**) or 10% (*).*
